# Pain Management Practices by Nurses: An Application of the Knowledge, Attitude and Practices (KAP) Model

**DOI:** 10.5539/gjhs.v8n6p154

**Published:** 2015-10-26

**Authors:** Bashar I. Alzghoul, Nor Azimah Chew Abdullah

**Affiliations:** 1 School of Business Management, Universiti Utara Malaysia (UUM), Kedah, Malaysia

**Keywords:** attitude, knowledge, nurses, pain management practices

## Abstract

Pain is one of the most common reasons that drive people to go to hospitals. It has been found that several factors affect the practices of pain management. In this regard, this study aimed at investigating the underlying determinants in terms of pain management practices. Based on reviewing the previous studies and the suggestions of the KAP model, it was hypothesized that the main elements of the KAP model (attitudes and knowledge) significantly predict the variation in the practices of nurses regarding pain management. A questionnaire comprising the KAP model’ s constructs, i.e. knowledge and attitude towards pain management, as well as pain management practices, was used to collect data from 266 registered nurses (n=266) who are deemed competent in the management of patients’ pain in the Jordanian public hospitals. The two constructs, attitude and knowledge, which are the main determinants of the KAP model were found to independently predict nurses’ practices of managing patients’ pain. Knowledge of pain management was found to be the strongest predictor. Additionally, it was found that about 69% of the variance in pain management could be explained by the constructs of the KAP model. Therefore, it is recommended that the Jordanian hospitals and universities focus on nurses’ knowledge and attitude towards pain management in order to enhance their practices in the field of pain management.

## 1. Introduction

Pain is one of the most common symptoms experienced by patients (Clinical Standards Advisory Group [CSAG], 1999), and approximately 79 % of hospitalized patients suffer from it (Lui, So, & Fong, 2008). Pain management practices are defined as a set of activities that should be provided by nurses to manage the patients’ pain effectively (Hossain, 2010). These activities include assessing the patients’ pain (Kwekkeboom & Herr, 2001), providing appropriate nurse’s interventions to relieve the patients’ pain (Summer & Puntillo, 2001) and reassessing the patients’ pain after intervention (Cullen, Greiner, & Titler, 2001). The pain management practices in any healthcare system were affected by three major barriers which include patients’ barriers, organizational barriers and healthcare providers’ barriers (Glajchen, 2001; Jacobsen et al., 2009).

Nurses are not the only healthcare providers who are responsible for relieving patients’ pain (McMillan, Tittle, Hagan, Laughlin, & Tabler, 2000), but they also play a key role in managing patients’ pain (Lewthwaite et al., 2011). This is attributed to the fact that the nurses are in a central position between the responsible physicians and their patients (McCaffery & Pasero, 1999; Schafheutle, Cantrill, & Noyce, 2001).

Neglecting patients’ pain is an unacceptable behavior ethically and morally (Gunningberg & Idvall, 2007; Royal College of Surgeons & College of Anaesthetists, 1990), and it leads to many consequences and complications for both the patients and the healthcare organizations (Hutchinson, 2007). Therefore, many international organizations concerned with improving the patients’ safety and healthcare quality, paid attention to this problem, reporting that the nurses provide inadequate pain management in all countries (The Agency for Health Care Policy & Research [AHCPR], 2002; The Joint Commission on Accreditation of Healthcare Organizations [JCAHO], 1999; The Oncology Nursing Society [ONS], 2012).

Studies conducted among Jordanian nurses indicated that nurses provide inadequate pain management. Daibes (2011) found that nurses did not perform pain management for their patients. In particularly, Daibes’s findings revealed that nurses in Jordan’s hospitals provide inadequate intervention to relieve the patients’ pain and they did not undertake any immediate action to manage the patients’ pain (Daibes, 2011). Another Jordanian study conducted by Abdalrahim, Majali and Bergbom (2008) aimed at assessing the nurses’ pain management practices. They found that the pain assessment scale was used by only 4.3 percent of Jordanian nurses.

The knowledge, attitude and practices (KAP) model is one of the most used models in the medical field. According to Launiala (2009), this model was first used during the middle of the nineteenth century to assess family planning and population (Launiala, 2009). The KAP model suggests that any practices (behaviors) are determined by the person’s attitude and knowledge towards the behaviors.

However, studies which assessed the relationship between the healthcare providers’ knowledge, attitude and their pain management practices have been neglected in the Middle East regions (Basak, 2010; Hossain, 2010). Therefore, the results of those studies may not correspond to the result in one of the Middle East countries (Jordan). Hence, the aim of this study was to identify the factors that influence nurses’ practices regarding pain management in Jordan.

## 2. Methods

A cross-sectional non-experimental survey design was used to determine the ability of the Knowledge, Attitude and Practice (KAP) model to predict nurses’ practices to manage the patients’ pain. The research received ethical approval from Universiti Utara Malaysia (UUM), Military Hospitals and the Jordanian Ministry of Health (JMoH) prior to data collection. Data collection was started on October 2014 and completed by March 2015. To facilitate the process of data collection, the questionnaires were distributed and received through the charge nurses for each shift. The sample of the current study were registered nurses (RN) (n = 266). These nurses have the following criteria. Their level of nursing education should be at least bachelor’s degree. They have their practicing license issued by the Nursing Council of Jordan, and they must also be full time employees at one of the public hospitals.

**Figure 1:**
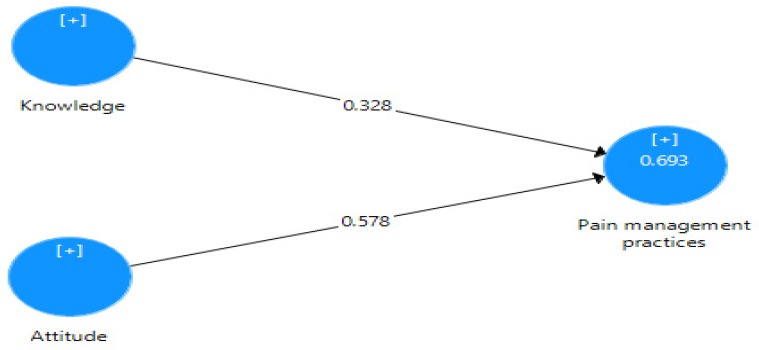
Measurement Model

This study is a correlational study. Consistent with the KAP Model, this study was conducted using a self-report questionnaire comprising 67 items. These items were classified into groups to reflect the three main constructs of Attitude, Knowledge and Pain Management Practices; Attitude was assessed using 22 items; and Knowledge of Pain Management with nine items on a seven-point Likert Scale ranging from “7 = strongly agree’’ to ‘‘1 = strongly disagree’’. The third construct, Pain Management Practices, was assessed using 36 items, using the seven-point Likert Scale, ranging from ‘‘7 = constantly to ‘‘1 = Never. Figure 1 shows the KAP Model, indicating the direct determinants of pain management practices, including attitude and knowledge of pain management.

The statistical results of the descriptive data (respondents’ information and questionnaire constructs) were obtained using the Statistical Package for Social Sciences (SPSS version 20), and the assessment of measurement model and structural model were obtained through Partial Least Squares-Structural Equation Modeling (PLS-SEM version 3.0).

## 3. Results

The majority of the respondents (160 nurses) in this study were female nurses (60.2%). More than half of the respondents belonged to the age group of 26-30 years old and the average nurses’ age was approximately 30 years. Also, the majority of the respondents (85 nurses) had professional experiences between 6 and 10 years (32 %). 231 (86.8%) respondents had a bachelor degree, whereas 19 of the respondents (7.1 %) had a master degree. Data indicated that the majority of nurses (195 nurses) had never attended a pain management training program (73.3 %). Finally, most of the nurses (210 nurses) mentioned that they had a pain experience in their life (78.9 %). Table 1 represents the demographic characteristics of the respondents.

**Table 1:** Demographic characteristics of the respondents

		Frequency	Percentage
Gender			
Male		99	37.2
Female Missing Values		160 7	60.2 2.6

Education			
Bachelor degree		231	86.8
Master degree Missing Values		19 16	7.1 6.0

Experience			
From 1-5 years		83	31.2
From 6-10 years		85	32.0
From 11-15 years		39	14.7
From 16-20 years Missing Values		28 31	10.5 11.7

Pain Experience			
Yes		210	78.9
No Missing Values		52 4	19.5 1.5

Training			
Yes		66	24.8
No Missing Values		195 5	73.3 1.9

Age	Missing Values	Mean	Std. Deviation
	39	30.30	5.809

As represented in Table 2 below, the mean and standard deviation for the nurses’ attitude towards pain management were 4.504 and 0.935, respectively. This suggests that the nurses tended to have moderate level of attitude towards pain management. Also, the results showed a moderate score for the nurses’ knowledge of pain management (Mean = 4.338, Standard deviation = 1.032). Additionally, this table showed that the nurses tended to have a moderate level of pain management practices (Mean = 4.968; standard deviation = 1.310).

**Table 2:** Descriptive statistics for latent variables

Latent Constructs	Number of Items	Mean	Std. Deviation
Attitude	22	4.504	0.935
Knowledge	9	4.338	1.032
Pain Management Practices	36	4.968	1.310

**Figure 2:**
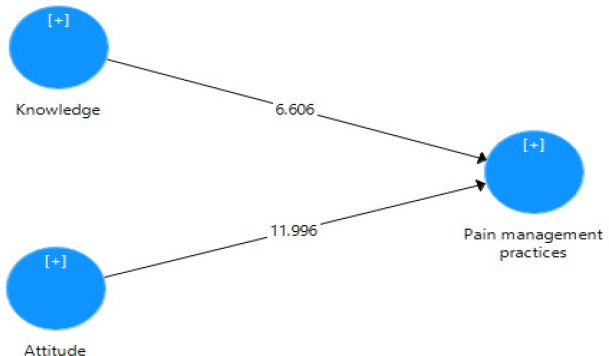
Structural mode

The significance of the path coefficients assessed in this study was measured using the standard bootstrapping procedure which includes 5000 bootstrap samples and 266 cases as recommended by Hair, Hult, Ringle and Sarstedt (2014). Attitude towards pain management and knowledge of pain management were both positively correlated with pain management practices. Specifically, the study results (Table 3 and Figure 2) demonstrated a significant positive relationship between the nurses’ attitude towards pain management and pain management practices (β = 0.578, t = 11.996, p< 0.001). Additionally, the study’s findings (Table 3, Figure 2) revealed that the knowledge of pain management had a strong association with pain management practices (β = 0.328, t = 6.606, p< 0.001).

**Table 3:** Structural model assessment

Hypotheses	Relationships	Beta	Std. Error	t- value	p-value
H1	Attitude Towards Pain Management	0.578	0.048	11.996	0.000***
H2	Knowledge of Pain Management	0.328	0.050	6.606	0.000***

*Note.* Endogenous Latent Construct = Pain Management Practices.

*** Significant at 0.01 (1-tailed),

**significant at 0.05 (1-tailed), *significant at 0.10 (1-tailed).

Based on the findings, the KAP Model’s constructs of Knowledge and Attitude are statistically significant and explained about 69 % of the variance in pain management practices (see Figure 1). The highest positive contribution in predicting pain management practices of the participants was made by Attitude towards pain management (b = 0.578, p < 0.001); whereas knowledge of pain management contributed significantly to pain management practices of nurses (b = 0.328, p < 0.001).

## 4. Discussion

The KAP model proposes that any practices are influenced by the two constructs of attitude and knowledge. In this study, the attitude towards pain management is defined as the general feeling of favorableness or unfavorableness toward performing pain management. Additionally, Alley (2001) defined the Knowledge of pain management as “Knowledge technologies used by nurses to help patients to achieve optimal pain relief”. According to Hossain (2010), pain management practices refer to the activities the nurses perform in order to relieve the patients’ pain. The KAP model suggests that people with a high positive attitude towards behavior and high knowledge will have an effective practice.

The findings showed that the nurses’ attitude towards pain management had a significant and positive relationship with their pain management practices in the Jordanian public hospitals. This finding seems to be consistent with Rony et al. (2010) who found a significant positive linking between the parents’ attitude towards pain management and their actual pain management practices. Also, the findings were consistent with Edwards et al. (2001) and Jurgens (1996) who found a significant and positive relationship between the nurses’ attitude towards pain management and their likelihood to administer analgesics. Similarly, the study’s findings indicated that that the relationship between knowledge and pain management practices was positively significant. This result is consistent with previous findings (e.g. Glajchen & Bookbinder, 2001).

The KAP Model has been utilized to assess the practices of health providers towards pain management practices (Basak, 2010; Hossain, 2010). Basak (2010) conducted a study of 100 nurses in Bangladesh and found that the KAP Model accounted for 16% of the variance in pain management practices. In addition, the findings indicated that attitude and knowledge are not significant within the model. Another study used the KAP Model to examine pain management practices of post-operative children by nurses (Hossain, 2010), utilizing a cross-sectional design to collect data from 93 pediatric surgical nurses to investigate the determinants of pain management practices. The findings of this study revealed that Attitude towards and Knowledge of pain management variables did not contribute significantly to pain management practices by the nurses (Hossain, 2010). Hence, the current study’s findings are not consistent with the two studies mentioned above. This indicates normative influences to be statistically insignificant predictors of pain management practices (Basak, 2010; Hossain, 2010). On the other hand, the findings of this study are consistent with the suggestions of the KAP Model.

This study is not without a few methodological limitations. These limitations must be noted as they can affect the generalization of the study, including: (i) the low response rate (only 51%); and (ii) the limited sample (266 registered nurses). Additionally, because this study is a voluntary survey, the results may be skewed and do not represent the views of all Jordanian nurses.

The KAP Model has proven to be a useful model to predict pain management practices of nurses from the perspective of attitude and knowledge. The implication of the findings is that interventions should focus on changing attitudes and improving knowledge in order to enhance pain management practices of nurses.

## 5. Conclusions

This study used the KAP Model to identify the factors that influence pain management practices of patients. The direct measures of the KAP Model explained 69% of the variance with regards to the practices of nurses to alleviate the pain suffered by their patients. The findings of this study provide theoretical support for using the KAP Model to study pain management practices among nurses. Nevertheless, it is recommended that further research should be undertaken to identify the factors that contribute to the remaining 31% of the variance in the management of pain by the nurses. It is therefore recommended that this study can be replicated using a larger population and different clinical practice regimes. Overall, this study shows that the attitude towards and knowledge of pain management of the nurses statistically and significantly contribute to pain management practices. Thus, the hospitals and universities in Jordan should focus on these factors to improve the nurses’ practices regarding pain management.
